# Assessing indications for herbal medicinal products: a comparative analysis of EMA monographs and database records

**DOI:** 10.1186/s12906-025-04852-8

**Published:** 2025-04-09

**Authors:** Hai Anh Nguyen, Jennifer Doerfler, Jens Buentzel, Christian Keinki, Jutta Huebner

**Affiliations:** 1 Klinik Für Innere Medizin II, Hämatologie Und Internistische Onkologie, Universitätsklinikum Jena, Friedrich-Schiller-Universität Jena, Am Klinikum 1, Jena, 07747 Deutschland; 2 Klinik Für HNO Erkrankungen, Kopf-Hals-Chirurgie, Südharz-Klinikum Nordhausen gGmbH, Nordhausen, Deutschland

**Keywords:** Herbal medicinal product, Phytopharmaceutical, EMA, Monograph, List entry, Integrative medicine, Cancer

## Abstract

**Background:**

Adverse effects are common during cancer treatment and herbal medicinal products (HMPs) are one way to manage symptoms caused by conventional therapy.

**Objectives:**

This assessment focused on comparing HMP indications listed in European Medicines Agency (EMA) monographs with findings in Medline and the Cochrane Library. The objective of this study was to investigate whether there is evidence that HMP indications may be transferred from non-cancer patients to cancer patients for the treatment of therapy-induced symptoms.

**Methods:**

This study design included a comprehensive review of the relevant literature.

**Results:**

The systematic literature search identified 96 clinical trials from a total of 726 records that met all the inclusion criteria. An analysis was performed on two groups: the EMA indication group vs. the non-EMA-indication group. The EMA indication group comprises trials whose endpoints align with the indications outlined in EMA monographs, representing a slight majority of 58.3% of all eligible clinical trials. In contrast, 41.7% of all studies were found to have non-EMA-indications, i.e. indications for cancer patients not listed in EMA monographs. Approximately 71.1% of all phytopharmaceuticals are approved as traditional use HMPs (THMPs).

**Conclusion:**

The efforts of the EMA represent a fundamental step toward securing the quality of HMPs in the European Union (EU). However, a more systematic approach to conducting studies in such a tradition-bound field is required to generate evidence on HMPs. Given the absence of sufficient data, it is not possible to make a definitive statement on the transferability of HMP scopes listed in EMA monographs to the management of treatment-related symptoms in cancer patients.

**Supplementary Information:**

The online version contains supplementary material available at 10.1186/s12906-025-04852-8.

## Introduction

### Herbal medicinal products

Herbal medicinal products were introduced in Directive 2004/24/EC of the European Parliament and Council as “any medicinal product, exclusively containing as active ingredients one or more herbal substances or one or more herbal preparations, or one or more such herbal substances in combination with one or more such herbal preparations” [1]. Overall, phytotherapeutics can be classified into two categories: 1) well-established use HMPs (WEU-HMPs) and 2) traditional use HMPs (THMPs).

Well-established use HMPs are plant-derived formulations whose safety and efficacy are substantiated by the scientific literature, and they have been used in the European Union for at least ten years. To be granted marketing authorisation, the evidence may be provided with at least one controlled clinical trial or well-documented clinical experience with sufficient supporting pharmacological data [1, 2].

In contrast, traditional herbal medicinal products undergo a simplified registration procedure. By demonstrating a traditional use record of at least 30 years, including a minimum of 15 years within the European Union, as well as providing sufficient data proving safety and plausible efficacy based on long-standing experience, the conduct of state-of-the-art clinical trials can be replaced. Moreover, THMPs intended for use without medical supervision must adhere to specified strength and posology guidelines, and be limited to oral, external, or inhalation preparations [1, 2].

### Committee on Herbal Medicinal Products (HMPC)

The Committee on Herbal Medicinal Products (HMPC) was established in September 2004 with the enforcement of Directive 2004/24/EC on Traditional Herbal Medicinal Products [1]. It is an independent committee of the European Medicinal Agency responsible for developing standardised criteria for the evaluation of herbal substances and preparations. The panel is composed of primary and substitute members appointed by EU member states, the countries of the European Economic Area (EEA) and the countries of the European Free Trade Association (EFTA). Additionally, up to five co-opted members are chosen based on their specialised expertise as nominated by Member States or the Agency when deemed necessary. The duration of their tenure spans a period of three years [1–3].

Scientific findings on HMP are summarised in monographs based on assessments of all data accessible to the public and information sourced from regulatory bodies across EU nations. Among other things, the quality, safety, and efficacy of various herbal medicinal products have been evaluated in order to harmonise their authorisation on the European market [1–5].

Previous studies have shown that most HMPs have been registered or licenced with corresponding EMA dossiers [6], indicating the importance of monographs as the means to facilitate authorisation procedures while securing high safety and efficacy standards. While monograph standards are recognised and implemented by the majority of EU member states [7], it is important to mention that these documents solely offer recommendations, meaning that they are not legally binding. Nonetheless, member states are required to provide appropriate reasons for rejecting the monographs and publicly state their dissenting opinions [3, 7, 8].

### Authorisation process

The authorisation procedure commences with a call for scientific data. The Monograph and List Working Party (MLWP) appoints a rapporteur to research clinical and nonclinical data on the HMP sought and compiles all the results in a monograph, a detailed assessment report and a list of references. Due to significant safety concerns or an insufficient amount of scientific data, the development of monographs may not be possible in some cases. In these situations, public statements are issued. The documents are discussed by the HMPC, and stakeholders have the opportunity to provide further information and state their opinions. The rapporteur summarises the results of the negotiation in a synopsis, assesses the need for further revisions and submits the result to the MLWP. Once the MLWP has reached an agreement on the finalisation of the document package, it is evaluated by the HMPC and formally adopted. All documents and dissenting opinions are published on the EMA website [3–5, 7, 8].

### Purpose of this systematic comparative analysis

The German S3 guideline “Complementary medicine in the treatment of oncological patients” is an extensive systematic evidence-based guideline that addresses the use of HMPs in cancer patients in a comprehensive chapter [9]. In this context, experts have discussed whether results obtained from studies examining the use of phytopharmaceuticals to alleviate particular symptoms in non-cancer patients can also be applied to cancer patients. For instance, research has been conducted to determine whether the use of black cohosh in the management of hot flashes can be extended to mitigate the same symptoms caused by hormone therapy in breast cancer patients [9]. This transfer demonstrates an important advancement as unfortunately, there is no evidence for the efficacy of most HMPs in the context of cancer. Furthermore, the S3 guidelines concluded that no recommendation could be made for the majority of herbs, as studies involving cancer patients are missing [9]. The objective of this comparative analysis was to investigate the potential transferability of HMP applications in the management of treatment-induced symptoms in patients with cancer. We conducted a structured assessment of the potential alignment between the indications of HMPs listed in EMA monographs and the endpoints reported in clinical trials, aiming to explore the influence of phytopharmaceuticals on the quality of life (QoL) in cancer patients. This focus was specifically chosen to address the considerable symptom burden and high prevalence of treatment-related adverse effects experienced by oncological patients undergoing surgery, chemotherapy and/ or radiotherapy [10, 11]. Symptoms such as fatigue and nausea often require effective supportive care, especially when conventional treatment is contraindicated, poorly tolerated or insufficient. Rossi et al. observed that 38.3% of cancer patients in Europe, as many as 50% in Italy, used HMPs for the management of adverse effects [10]. The use of HMPs among cancer patients may increase over the course of their illness. Molassiotis et al. reported a notable rise in the administration of HMPs from 5.3% prior to diagnosis compared to 13.9% afterward, representing an increase of approximately threefold [12]. By examining the reduction of adverse effects and the improvement of QoL, the transferability of HMPs from non-cancer to cancer care can be assesses not only through clinical outcomes but also by evaluating their impact on patients’ overall experience, physical functioning and mental health within a patient-cantered framework. The transferability is relevant across all types, stages and treatment phases of cancer, making the results broadly applicable. By confining the investigation to this clinically relevant population, our comparative analysis assesses the potential of HMPs as a complementary, personalised and safe approach for managing therapy-induced symptoms. The identification of overlaps, gaps and inconsistencies between EMA monographs and clinical data will contribute to evidence-based decision-making in integrative oncology and enhance supportive care strategies for cancer patients.

## Material and methods

To identify relevant literature for this review, a comprehensive search was conducted in two established databases for medicine and healthcare-related topics: Medline and the Cochrane Library. Data collection was performed from December 2023 to April 2024. The Medical Subject Heading (MeSH) term “neoplasm” was combined with the Latin or the English common name of the HMP, which were explored within the title or abstract using the field code limiter [Title/Abstract] in Medline and [Title/Abstract/Keyword] in the Cochrane Library, respectively. The search algorithm is described as follows:

- Medline: neoplasm[MeSH Terms] AND ((Latin Name of herbal substance[Title/Abstract]) OR (English common name of herbal substance[Title/Abstract])) Filters: Clinical Trial
- Cochrane Library: neoplasm[MeSH Terms] AND ((Latin Name of herbal substance[Title/Abstract/Keyword]) OR (English common name of herbal substance [Title/Abstract/Keyword]))

Search results from both databases were incorporated into a combined EndNote library, with duplicate entries carefully removed. Based on predefined inclusion and exclusion criteria, the retrieved publications were evaluated for eligibility by two independent reviewers (HN, JH). The abstracts were screened first, followed by a full-text search to assess all potentially relevant references and evaluate whether they meet the criteria for inclusion. Disagreements about study eligibility were resolved by discussion and consensus.

For this review, only randomised controlled trials (RCTs) were deemed eligible for inclusion, encompassing both parallel group and crossover studies, since RCTs offer a low susceptibility to bias and allow a powerful causal examination of cause and effect. Clinical trials investigating patient-relevant outcomes (PROs) such as treatment-related adverse effects or QoL, were analysed for individuals receiving chemotherapy and/or radiotherapy as part of their confirmed oncological disease. These patients represent a vulnerable population with complex medical needs, making it crucial to explore supportive therapeutic options to manage the adverse effects of cancer and improve overall well-being.

In the enrolled clinical trials, the intervention group may have received any herbal medicinal product described in the final European Union herbal monographs and list entry catalogue used as a stand-alone ingredient with a valid EMA monograph. Conversely, the control group shall be assigned to a placebo, no treatment, or another active intervention.

Cellular experiments and trials involving healthy subjects as well as patients without confirmed cancer diagnoses were excluded from this review. HMPs without an EMA monograph were also eliminated as they could not be published due to safety concerns or a lack of scientific evidence, as mentioned above. Furthermore, HMPs that were administered in combination with other products or had not been examined in a separate experimental arm were excluded because their individual impact cannot be adequately assessed. Unfinished trials registered on clinicaltrials.gov that did not have a final publication were also not included. Studies that aimed to prevent cancer or detect precancerous changes were excluded as no proven cancer was diagnosed in these patients.

A comprehensive analysis was performed to evaluate the therapeutic use of HMPs as outlined in EMA monographs in comparison to those reported in clinical trials. All indications for both WEU-HMPs and THMPs described in EMA monographs were carefully identified and catalogued. From all RCTs included in review, relevant endpoints were extracted focusing on outcomes that measure the impact of HMPs on the QoL and management of adverse effects. A systematic mapping process was initiated to ensure the alignment between the reported indications. For example, if the EMA document specified the use of *ginger* for “nausea and vomiting in motion sickness” endpoints such as “chemotherapy induced nausea” were compared to this indication. Discrepancies between monograph indications and study findings were documented. To ensure accuracy, the data extraction process for both EMA indications and study endpoints was performed independently by HN and JH, with discrepancies resolved through consensus.

To assess the efficacy of HMPs for symptom management in cancer patients, *p*-values from primary and secondary endpoints were systematically analysed across 96 clinical trials. Unless otherwise specified, an outcome was considered significant if *p* < 0.05. In studies with multiple endpoints, the use of HMP was evaluated as effective if the majority of the outcomes demonstrated statistical significance. Using predefined Excel formulas, the overall significance was calculated, and trials were categorised into three groups: 1) effective HMPs 2) ineffective HMPs and 3) HMPs with unclear efficacy.

## Results

**Fig. 1:**
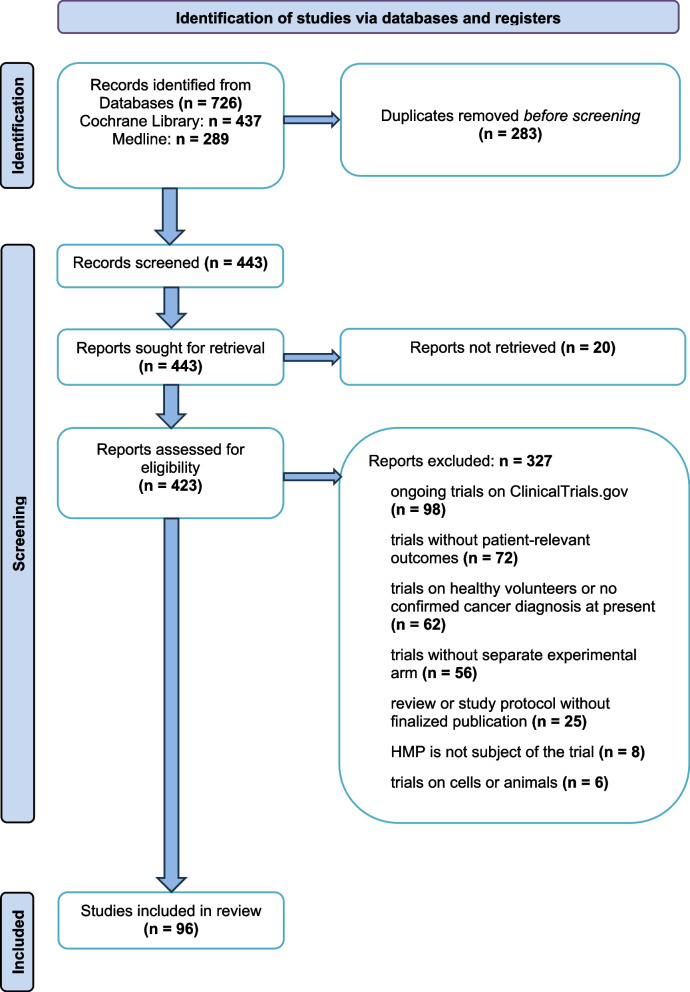
PRISMA Flow Diagram

**Fig. 2:**
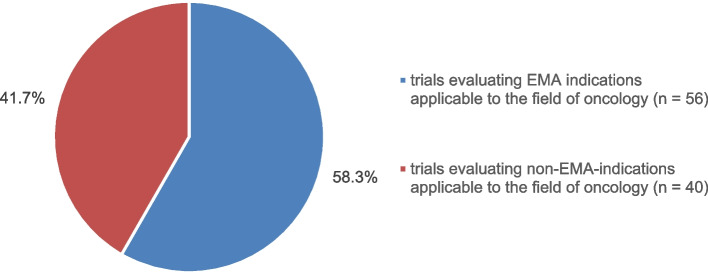
Distribution of Eligible Clinical Trials

Based on the table published by the European Medicines Agency on 4 December 2023 [13], a total of 194 herbal medicinal products were examined after excluding one duplicate and combined preparation (*n* = 7). A comprehensive literature search according to the previously described algorithm resulted in a total of 726 potential studies. Figure 1 illustrates the process of identifying, screening, and selecting suitable clinical trials. After removing duplicates (*n* = 283) and non-retrievable references (*n* = 20), 423 records remained for full-text assessment. A total of 327 search results were considered unsuitable because they did not meet the inclusion criteria. Ultimately, 96 clinical trials were deemed eligible for inclusion, with 56 studies evaluating at least one EMA indication and 40 studies examining endpoints other than those specified by the EMA, also known as non-EMA-indications. For the selection of the studies, see Fig. 2 and additional files 1, 2, 3 and 4.

### Evaluation of THMP and WEU-HMP Distribution

**Fig. 3:**
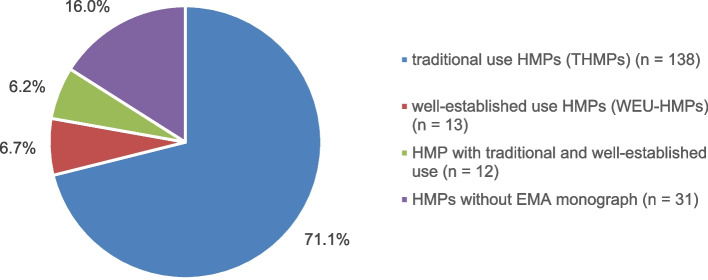
Distribution of THMPs and WEU-HMPs

Of the total of 194 phytopharmaceuticals, 138 are HMPs with traditional use, which are subject to a simplified authorisation procedure based on longstanding use. THMPs represented the largest proportion of all plant-based preparations analysed, accounting for 71.1% as shown in Fig. 3. This finding is consistent with the results presented by Peschel et al., who reported that the majority of herbal medicines are registered as THMPs [6]. Only a small fraction of 6.7% comprises well-established use HMPs which are subject to a more stringent approval process to obtain marketing authorisation as clinical trials must comprehensively prove their safety and efficacy. Furthermore, there are phytopharmaceuticals approved for both traditional and established use, representing 6.2% of all HMPs examined. Ultimately, 16% of all HMPs do not have a valid monograph and therefore cannot be assigned to either THMPs or WEU-HMPs. Further details can be found in additional file 3.

### EMA indication group

Fifty six clinical trials, representing 58.3% of all included studies, evaluated at least one EMA indication with the majority of them being investigated as a primary endpoint. Consequently, EMA indications align with the endpoints of these studies and can be assessed for the relief of symptoms caused by cancer treatment. The following HMPs were investigated in the context of oncological diseases as shown in Table 1 as well as additional file 1 and 2: *black cohosh* was utilised for the management of hot flashes [14–17], *centella* for mitigating radiation dermatitis [18], *cranberry* for alleviating urinary tract symptoms [19–22], *ginger* for ameliorating chemotherapy induced nausea and vomiting [23–42], *lavender flower/ lavender oil* for reducing stress and improving sleep quality [43–49], *linseed* for addressing symptoms of radiation esophagitis [50, 51] and *roman chamomile flower* for relieving gastrointestinal complaints [52]. In addition, *ginseng* [53–61], *green tea* [62–65], and *guarana* [66–69] can be used to combat fatigue. EMA indications were mostly addressed as primary endpoints with the exception of eight studies [28, 41, 42, 48, 51, 62, 63, 69].

**Table 1:** Studies Evaluating EMA Indications Applicable to the Field of Oncology

**application in cancer patients**	patients with breast cancer dealing with hot flashes	patients with breast cancer dealing with radiation dermatitis	patients with prostate, bladder or cervix cancer dealing with radiation cystitis and other urinary tract symptoms	patients with different types of cancer dealing with chemotherapy induced nausea	patients with different types of cancer dealing with fatigue	patients with different types of cancer dealing with fatigue	patients with different types of cancer dealing with fatigue	patients with different types of cancer dealing with anxiety and insomnia	patients with non-small cell lung cancer dealing with radiation esophagitis	patients with different types of cancer dealing with abdominal pain, obstipation, or diarrhoea
EMA indications	menopausal complaints such as hot flashes and profuse sweating in breast cancer patients	healing of minor wounds	mild recurrent lower urinary tract infections such as burning sensation during urination and/or frequent urination	• nausea and vomiting in motion sickness • mild, spasmodic gastrointestinal complaints including bloating and flatulence	symptoms of asthenia such as fatigue and weakness	fatigue and sensation of weakness	fatigue and sensation of weakness	mild symptoms of mental stress and exhaustion and to aid sleep	• mild gastrointestinal discomfort • habitual constipation or in conditions in which easy defaecation with soft stool is desirable	mild, spasmodic gastrointestinal complaints such as bloating and flatulence
references	Jacobson [14], Pockaj [15], Pockaj [16], Rostock [17]	Thanthong [18]	Campbell [19], Cowan [20], Hamilton [21], Herst [22]	Ansari [23], Arslan [24], Bossi [25], Bhargava [41], Crichton [26], de Queiroz [42], Evans [27], Khiewkhern [28], Konmun [29], Li [30], Lua [31], Manusirivithaya [32], Marx [33], Panahi [34], Pillai [35], Ryan [36], Sanaati [37], Santos [38], Thamlikitkul [39], Williams [40]	Barton [53], Barton [54], Guglielmo [55], Hamidian [56], Jiang [57], Kim [58], Kim [59], Yennurajalingam [61], Yennurajalingam [60]	Liu [62], Choan [63], Jatoi [64], Pisters [65]	da Costa Miranda [66], de Oliveira Campos [67], del Giglio [68], Palma [69]	Beyliklioğlu [43], Khamis [44], Kohara [49], Ozkaraman [45], Şahin [46], Shammas [47], Soden [48]	Andersson [50], Lim [51]	Wilkinson [52]
number of clinical trials	**4**	**1**	**4**	**20**	**9**	**4**	**4**	**7**	**2**	**1**
HMP	**black cohosh**	**centella**	**cranberry**	**ginger**	**ginseng**	**green tea**	**guarana**	**lavender flower/ lavender oil**	**linseed**	**roman chamomile flower**

### Non-EMA-indication group

In contrast, 40 studies examined endpoints other than those specified by the EMA. This group, representing 41.7% of all eligible clinical trials, explored fields of application for cancer patients not listed in EMA monographs, known as non-EMA-indications as shown in Table 2, additional files 1 and 2.

**Table 2:** Studies Evaluating Non-EMA-Indications Applicable to the Field of Oncology

**Non-EMA-indications applied in cancer patients**	• chemoradiotherapy induced oral mucositis • radiation dermatitis • radiation proctitis • cancer progression	stabilise liver function	fever	• menopausal symptoms • QoL • cancer progression	• chemoradiotherapy induced oral mucositis • cancer recurrence	odour management of colostomy bag	nausea, vomiting and retching	radiation induced lung toxicity	• chemoradiotherapy induced oral mucositis • radiation dermatitis • nausea and vomiting	anxiety and sleep quality	• chemoradiotherapy induced oral mucositis • radiation dermatitis • QoL	fatigue
EMA indications	occasional constipation	relief of itching in acute and chronic dry skin conditions	• common cold • atherosclerosis	symptoms of asthenia such as fatigue and weakness	fatigue and sensation of weakness	mild symptoms of mental stress and exhaustion and to aid sleep	• minor spasms of the gastrointestinal tract, flatulence, and abdominal pain, especially in patients with irritable bowel • mild tension type headache • cough and cold • localised muscle pain • • • localised pruritic conditions in intact skin	occasional constipation	mild, spasmodic gastrointestinal complaints such as bloating and flatulence	• treatment of small superficial wounds and insect bites • treatment of small boils (furuncles and mild acne) • relief of itching and irritation in cases of mild athlete foot • treatment of minor inflammation of the oral mucosa	digestive disturbances, such as feelings of fullness, slow digestion, and flatulence	• temporary loss of appetite • mild, spasmodic gastrointestinal complaints • small superficial wounds • minor spasm associated with menstrual periods
references	Alkhouli [70], Heggie [71], Hoopfer [72], Lissoni [73], Lissoni [74], Momm [75], Nyström [76], Sahebjamee [77], Sahebnasagh [78], Sahebnasagh [79], Su [80], Tungkasamit [81], Williams [82]	van der Merwe [83]	Gatt [84]	Chung [85], Kim [86], Xie [87]	Liao [88], Trudel [89]	Duluklu [90]	Efe Ertürk [91]	Yu [92]	Dos Reis [93], Ferreira [94], Garbuio [95], Maiche [96], Sanaati [37], Williams [40]	Ozkaraman [45]	Howells [97], Kia [98], Palatty [99], Rao [100], Ryan Wolf [101], Shah [102], Soni [103], Thomas [104]	Foucré [105], Ghadjar [106]
number of clinical trials	**13**	**1**	**1**	**3**	**2**	**1**	**1**	**1**	**6**	**1**	**8**	**2**
HMP	**aloe vera**	**evening primrose oil**	**garlic**	**ginseng**	**green tea**	**lavender flower/ lavender oil**	**peppermint oil**	**rhubarb**	**roman chamomile flower**	**tea tree oil**	**turmeric**	**yarrow**

The EMA lists digestive disorders, such as bloating, slow digestion and flatulence, as possible fields of application for *turmeric* for which no clinical trials were found. However, *turmeric* is known for its anti-inflammatory properties, which is why five trials have investigated its effect on oral mucositis [98, 100, 102–104] and two trials have examined its impact on radiation dermatitis [99, 101]. Based on our findings, three clinical trials were published prior to 2019 [99–101], which is the year of publication of the EMA monograph on *turmeric*. Although these studies have demonstrated the safety and efficacy of *turmeric* in preventing dermatitis and mucositis, none of these indications were acknowledged in the monograph.

Our review did not uncover any clinical trials assessing the efficacy of *aloe vera* on occasional constipation. The most common endpoints were dermatitis, oral mucositis and proctitis following radiotherapy and/or chemoradiotherapy, which are probably not covered by the associated monograph as the data are inconclusive. Approximately half of the clinical trials analysed indicated the efficacy of *aloe vera* in soothing treatment-related inflammatory lesions [70, 75, 77–79, 81], whereas the remaining half reported no significant effects [71, 72, 76, 80, 82].

The effects of *peppermint oil*, *rhubarb* and *yarrow* on gastrointestinal complaints have not been investigated as intended by the EMA. However, their impact on nausea [91], radiation-induced lung toxicity [92] and fatigue [105, 106] have been reviewed.

In addition, further non-EMA-indications have been studied, including *evening primrose oil* to improve liver function [83], *tea tree oil* to manage anxiety and sleep disorders [45] as well as *garlic* to relieve fever [84]. The former two HMPs were intended to mitigate itching and dermal irritation while *garlic* was supposed to treat the common cold.

According to EMA monographs, *ginseng*, *green tea*, and *lavender flower/ lavender oil* are intended to treat symptoms of asthenia such as fatigue and weakness. In the case of *ginseng*, there are three clinical trials evaluating non-EMA-indications, one on menopausal symptoms [85], one on QoL [86] and one on cancer progression [87]. Moreover, two clinical trials on *green tea* have inspected oral health [88] and the prevention of cancer recurrence [89], both of which are beyond the indications outlined by the EMA. One study investigated ostomy odour reduction [90] as an additional use of *lavender flower/ lavender oil*.

*Studies on roman chamomile flower* display a high prevalence of non-EMA-indications. While the common cold was not assessed in any of the included studies, six clinical trials focused on radiation dermatitis (*n* = 3) [94–96], oral mucositis (*n* = 1) [93], and nausea (*n* = 2) [37, 40] as endpoints. Although there are data supporting the use of *roman chamomile flower* to relieve inflammation of the mouth and throat, it cannot be authorised as a THMP due to the record of use for this specific field of application being less than 30 years as requested per Directive 2004/24/EC [107].

### Analysis of geographical distribution

**Fig. 4:**
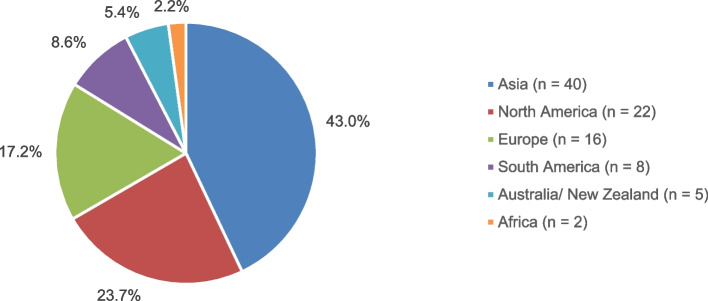
Geographical Distribution of Research Activity

The highest research activity was observed in Asia which contributed 43% of the eligible clinical trials in this survey as shown in Fig. 4 and additional file 3. One of the most notable research sites is Iran, which has conducted eight interventional studies [23, 34, 37, 56, 77–79, 98], making it the leading Asian country in terms of study count based on our search algorithm. India [35, 99, 100, 102–104], Thailand [18, 28, 29, 32, 39, 81], and Turkey [24, 43, 45, 46, 90, 91] are the countries with the third-highest number of surveys conducted, with each contributing six studies. This considerably promotes the scientific assessment of the safety and efficacy of HMPs such as *ginger* and *turmeric* which are becoming increasingly important in the EU.

Approximately 23.7% of all relevant assessments on HMPs were performed in North America, with the United States showing the highest overall research activity in the field of phytopharmaceuticals accounting for a total of 17 publications. Most frequently, the effects of *ginseng* on fatigue (*n* = 4) [53, 54, 60, 61], *black cohosh* on hot flashes (*n* = 3) [14–16] and *ginger* on nausea (*n* = 3) [27, 36, 40] were investigated with study endpoints aligning with indications specified by the European Medicines Agency. No indications beyond the predefined scope were explored in this context.

This analysis revealed that only 17.2% of the identified trials were undertaken in Europe with Germany [17, 75, 105, 106], Italy [25, 55, 73, 74], and the UK [20, 48, 52, 97] being the most engaged countries, with four clinical tests each. In addition, ten out of 16 studies published more than a decade ago indicated that the feasibility of research on HMPs is decreasing.

Finally, 8.6% of the studies were carried out in South America, 5.4% in Australia/ New Zealand and 2.2% in Africa.

### Efficacy of reviewed HMPs

**Fig. 5:**
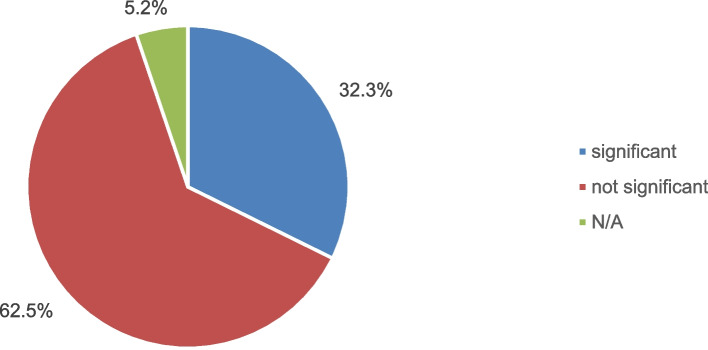
Efficacy of Reviewed HMPs

The efficacy of each HMP within its respective application domain was evaluated in additional file 4 through a *p*-value analysis of individual endpoints and overall statistical significance. Among 96 included clinical trials, 32.3% reported statistically significant effects in their primary and secondary outcomes, while the majority of 62.5% found non-significant results, and 5.2% did not provide sufficient data (absence of *p*-value) to determine significance as shown in Fig. 5. Non-significant outcomes were predominant in both groups, although they were slightly higher in the non-EMA-indication group (70% vs. 57.1%). Additionally, the EMA-indication-group had a higher proportion of significant results (35.7%) compared to the other group (27.5%).

*Peppermint oil* and *tea tree oil* were identified as effective HMPs, showing statistically significant results in relevant studies. A clinical trial conducted by Efe Ertürk in 2021 demonstrated a notable reduction in the severity of nausea across an array of chemotherapy regimens, with the exception of cisplatin, when *peppermint oil* was employed as an adjunct therapy [91]. The administration of *tea tree oil* was found to enhance sleep quality and mitigate anxiety in cancer patients undergoing chemotherapy, as observed in the study performed by Ozkaraman [45]. These findings suggest that both HMPs may complement cancer treatment. However, as each HMP is supported by a single study, the existing evidence is insufficient to make conclusive recommendations.

The group of ineffective HMPs include *centella*, *cranberry*, *evening primrose oil*, *garlic*, and *rhubarb*. The currently available evidence does not support the efficacy of *centella* for the treatment of radiation dermatitis [18], *cranberry* for addressing prostatic issues [19] and urinary tract infections [20–22], *evening primrose oil* for improving liver function [83], *garlic* for reducing febrile neutropenia [84], and *rhubarb* for alleviating radiation-induced lung toxicity [92]. It is not advisable to administer this group of HMPs to cancer patients.

The majority of analysed HMPs demonstrate ambiguous efficacy, including those with indications outlined in EMA monographs. Despite expectations of proven therapeutic benefit, phytopharmaceuticals in the EMA indication group show data with both significant and non-significant results, highlighting a contentious reliability of EMA monographs. This group of herbal preparations with unclear efficacy consists of *aloe vera*, *black cohosh*, *ginger*, *ginseng*, *green tea*, *guarana*, *lavender flower/ lavender oil*, *linseed***,** *roman chamomile flower*, *turmeric*, and *yarrow*.

Overall, 61.5% of the studies on *aloe vera* were not significant. The findings indicate that *aloe vera* is ineffective in the treatment of radiotherapy-induced dermatitis [71, 72, 76, 81, 82]. Two studies verified the efficacy of *aloe vera* in alleviating oral mucositis [70, 75], while two other clinical trials did not confirm any such effect [77, 80]. Furthermore, Sahebnasagh demonstrated the anti-inflammatory properties of *aloe vera* in pelvic cancer patients in 2020 [78], but a subsequent study conducted in 2022 on colorectal cancer patients found no significant results in relieving proctitis [79].

The EMA has approved the use of *black cohosh* for the management of menopausal symptoms. It was found to reduce the frequency and intensity of hot flashes in a study conducted by Rostock [17], but this effect could not be observed by others [14, 15].

*Ginger* reduced chemotherapy-induced nausea in six studies [24, 29, 35–37, 41], aligning with EMA monographs. Nevertheless, the results of eleven trials [23, 25, 27, 28, 30–32, 34, 38–40] involving patients with different types and stages of cancer found *ginger* ineffective in managing nausea. While Crichton [26] reported significant improvements in nausea-related QoL, de Queiroz [42] found no effect. In summary, 35% of the clinical trials on ginger yielded significant results, 60% did not demonstrate efficacy, and 5% could not be categorised due to the absence of *p*-values in one study.

EMA monographs recommend *ginseng* for the management of fatigue. However, only two studies [57, 60] confirmed its efficacy in reducing exhaustion, while five [53–55, 59, 61] found no significant impact. Furthermore, an improvement in health-related QoL was observed [56, 58], although no effect on cancer remission was reported [87].

One study has verified the efficacy of *green tea in* combating fatigue [62], as recognised by the EMA. However, *green tea* has not been shown to improve oral health [88] or exhibit antineoplastic properties to prevent cancer progression [63, 64, 89].

The EMA monograph for *guarana* outlines its use in alleviating sensations of weakness. While de Oliveira Campos [67] reported a beneficial effect of *guarana* on fatigue, subsequent studies [66, 68, 69] found no evidence of efficacy.

The EMA approves *roman chamomile flower* for mild gastrointestinal complaints, as confirmed by Wilkinson's study [52]. In contrast, it showed no efficacy for non-EMA indications such as oral mucositis [93], radiation dermatitis [94–96], and nausea [37, 40]. All in all, 85.7% of the outcomes for this HMP were not statistically significant, while 14.3% were significant.

The results for *turmeric*, classified in the non-EMA-indication group, are inconclusive. A study conducted by Palatty [99] found that *turmeric* alleviated radiation dermatitis, while another by Ryan Wolf [101] showed no significant benefit compared to placebo in breast cancer patients. Similarly, *turmeric* reduced oral mucositis in two studies [100, 104] but did not provide a significant improvement in three other trials [98, 102, 103]. Additionally, no improvements in quality of life were observed [97].

The EMA recommends *yarrow* for managing fatigue. While Ghadjar [106] did not observe statistically significant effects on fatigue, Foucré [105] did not report a p-value, preventing further conclusions.

Although the EMA suggests that *lavender* may be beneficial in mitigating mental stress, fatigue and insomnia, clinical studies have revealed conflicting results regarding its efficacy. A total of 62.5% of the studies reported statistically significant results, whereas the remaining 37.5% showed non-significant findings. Efficacy was observed for anxiety [43, 45], fatigue [49], and sleep quality [44, 45]. Other studies reported that *lavender* had no significant stress-relieving and sleep-promoting effect [46–48].

The use of *linseed* was found to significantly alleviate mouth dryness [50], aligning with the EMA indication of mild gastrointestinal discomfort. In the absence of p-values, the efficacy of *linseed* against radiation esophagitis cannot be determined [51].

## Discussion

The slight majority of clinical trials, approximately 58.3% of all eligible studies, demonstrated that the endpoints assessed in the studies evaluating EMA indications are consistent with the fields of application presented in EMA monographs. This shows that documents published by the European Medicinal Agency provide a strong scientific foundation for the authorisation and regulation of phytopharmaceuticals. Furthermore, they promote the harmonisation of European standards on the safety and efficacy of herbal medicinal products.

While the outlook is promising, it remains a Sisyphean task to secure high-quality standards in the field of HMPs for the management of symptoms in patients undergoing conventional cancer treatment. By comparing the indications listed in the EMA monographs with the search results, this review sought to determine whether researchers investigating phytopharmaceuticals take EMA monographs into account when identifying potential areas of use for HMPs in patients with cancer. Although EMA monographs offer a valuable framework for the use of plant-based preparations in medical practice, researchers seem to explore different indications when planning studies in oncology settings. Moreover, the results of studies assessing indications from EMA monographs provide evidence that the traditional indications may be transferred to cancer care. This will be discussed in more detail below.

### Discrepancies Arising from Research

Researchers in the field of phytopharmaceuticals may overestimate the systematic approach to conducting studies on HMPs.

The field of herbal medicinal products is deeply rooted in cultural traditions which may influence the beliefs and methods of researchers to a great extent. Cultural bias might generate a greater degree of confidence in the efficacy of THMPs than the current evidence suggests. This phenomenon is reflected by the simplified authorisation procedure as THMPs are registered based on their long-term use of more than 30 years within the European Union [1, 2]. Overall, THMPs accounted for 71.1% of all HMPs evaluated, showing that beliefs and customs have a considerable influence on society. The concept of traditional use is not unique to Europe but is similarly implemented in other parts of the world. In the US, the Dietary Supplement Health and Education Act of 1994 permits HMPs to be approved as dietary supplements through a simplified authorisation procedure [108]. At an individual level, researchers may be influenced by their own cultural biases. Scientists may identify areas of application for HMPs without relying on EMA monographs and other guidelines. Suggestions for using plant-based preparations may come from various sources such as a family’s repertoire of home remedies passed down through generations, different types of magazines or recommendations from healthcare professionals [109]. In this case, the study starts with the researcher’s personal experiences and convictions which are based on traditional use and anecdotal evidence leading to the investigation of indications not listed in EMA monographs. Personal experiences sometimes have a greater impact on the study design than the existing literature. According to a trial conducted in Bahrain, nearly 50% of the participating physicians acquired their knowledge of HMPs through experience rather than through scientific bibliography [110]. In our review, *yarrow* was assessed for its potential to alleviate fatigue despite unconfirmed anthroposophic medicine theories and a lack of clinical trials [105, 106]. Using phytopharmaceuticals based on their tradition might also predispose researchers to confirmation bias where they unconsciously seek out evidence that supports their pre-existing hypotheses and alter their approach to studies. This may result in arbitrary decisions regarding the sample size, the selection of participants for the control group or the use of subjective outcome measures.

Insufficient knowledge about or compliance with HMPs may also lead to the conduct of clinical trials, although EMA monographs have expressed concerns about the use of certain HMPs. Clement et al. conducted a study in which 60% of healthcare professionals believed in the effectiveness of HMPs in promoting general health [111], despite having limited knowledge of their associated risks and benefits [111–113]. Even in the absence of supporting evidence, the strong belief in the efficacy of certain HMPs can lead to their ongoing use without their legitimacy being questioned.

Investigating herbal medicinal products poses significant financial challenges. Specialised equipment and trained personnel are required to prepare plant-based preparations according to precise protocols. Additionally, expenses incurred for the employment of healthcare professionals and study assistants. Fees for the care and monitoring of patients must also be considered, among other expenditures. The availability of simplified authorisation as THMP allows pharmaceutical companies to circumvent the conduct of costly studies and reduce research funds on herbal medicine. When faced with limited funding, researchers may prioritise cost-saving measures and opt for streamlined methodologies that do not fully align with established guidelines. This can result in shortcuts or compromises in research practices, leading to discrepancies between EMA monographs and search results. The oral application of *aloe vera* to relieve occasional constipation as proposed by the EMA has been modified. Instead, commercially available *aloe* creams and gels are applied externally to reduce radiation-induced dermatitis [71, 72, 76, 81, 82] and oral mucositis [70, 77, 80]. A study conducted by Momm et al. tested Aldiamed gel, which is currently sold in German pharmacies for less than ten euros, for the treatment of oral mucositis [75]. As it is a ready-to-use product, factors such as cost reduction, standardisation of interventions and convenience support the feasibility of the trial.

### Discrepancies arising from EMA monographs

The peculiar regulations of EMA documents may also lead to differences in the scope of HMPs. One possible reason for the range of varying indications might be the safety and efficacy of herbal preparations. If a phytopharmaceutical is found to have adverse effects on health, HMPC may decide to not approve its use [1, 2]. Conversely, an HMP cannot be listed by the EMA if its efficacy for an indication is not sufficiently proven [1, 2]. As a result, the indication will not be included in the relevant monograph. The contradictory evidence for the efficacy of HMPs is illustrated by the example of *aloe vera*. Approximately half of the clinical trials reviewed suggest that *aloe vera* is effective in reducing treatment-related inflammation [70, 75, 77–79, 81], while the other half did not report any significant effects [71, 72, 76, 80, 82].

In addition, the clinical trials listed may not meet the regulatory requirements necessary for EMA approval. Possible reasons include small sample sizes, formulations deviating from EMA posology, or differences in the administration of the drug. According to EMA monographs, *roman chamomile flower* can be administered orally as an herbal tea or liquid preparation to relieve mild spasmodic gastrointestinal complaints. However, it was predominantly used as a topical gel or cream to treat radiation dermatitis. Additionally, doses not listed in the monographs were used.

Differences in the data may also be related to the population that was studied. As previously described in other studies, there is less information available for specific groups of patients than for the healthy population where HMPs are mainly used as a complementary tool to alleviate minor ailments intended to be used for a restricted period of time [4–8]. Cancer patients are a particularly vulnerable group. Interactions between ongoing oncological therapy and HMPs may occur, the mechanisms of which have not yet been sufficiently clarified. Instead, established pharmaceutical products whose safety, efficacy and range of side effects are already known might be favoured in such situations. For instance, laxatives rather than *aloe vera* may be used to treat occasional constipation. It is possible that there are a number of symptoms that can be addressed with HMPs in healthy individuals, but their use cannot be applied to cancer patients.

Furthermore, the authorisation of THMPs is susceptible to societal differences as knowledge of HMPs can vary culturally and geographically making it difficult to apply regulations to other regions and populations. Although EMA monographs serve as a means of harmonising European standards, it remains challenging for the HMPC to adopt these documents when the scope of HMPs differs locally. This is particularly relevant for HMPs that are not of European origin. *Turmeric* is commonly used in Europe for the treatment of digestive disorders, particularly bile duct conditions. In contrast, studies conducted in India have investigated the anti-inflammatory effects of *turmeric* on skin and mucous irritation, known as Kustha, in accordance with the Ayurvedic Pharmacopoeia of India [99, 100, 102–104, 114]. This example demonstrates that the HMPC does not recognise all possible therapeutic opportunities of HMPs, even though their safety and efficacy profile exceeds the indications listed by the EMA. The adjunct assessment report often provides insight into the evaluation process by providing information on the use of HMPs both inside and outside the EU [115]. Finally, the EMA monograph, the central reference of our review, represents only the final result adopted by the European Union.

The discrepancies observed can, to some extent, be attributed to the reactive approach taken by the HMPC in data collection for updating EMA monographs. These are subject to periodic review, either scheduled at five-year intervals or unscheduled at other times when new, pertinent findings emerge and are submitted to the HMPC for evaluation [116]. However, the HMPC does not actively gather information on HMPs, but rather issues a call for scientific data, inviting interested parties such as EMA members, industry players, research organisations or government agencies to submit new relevant evidence to revise monographs and supporting documents [117]. The impact of this reactive approach is evident in the gap between EMA indications and research findings. Our search identified seven studies on *turmeric’s* anti-inflammatory effects published between 2014 and 2023 [98–104], yet all monographs list it solely for treating digestive disorders [118, 119]. The first monograph, issued in 2009 [118], predated any of these studies. A call for scientific data in 2014 yielded no new findings, leaving the original monograph unchanged until 2018 [119]. By then, three studies [99–101] on *turmeric’s* soothing effects were available, but none were included in the list of references supporting the assessment report [120]. With at least four studies [98, 102–104] published since the 2018 revision, the anti-inflammatory properties of *turmeric* may be included in the next monograph, provided that studies meeting HMPC requirements are submitted. The absence of inflammation related indications for *turmeric* in the current EMA monograph may result from several factors, including insufficient reporting between researchers and the HMPC. International cooperation can be particularly challenging as the EMA, a European institution, operates within a confined geographic scope [1, 2]. Studies on *turmeric* were conducted in India [99, 100, 102–104], Iran [98] and the USA [101], which may lead researchers to prioritise local reporting over submitting their findings to Europe. Although regular international submissions to support the HMPC are crucial, factors such as time constraints, limited resources, and bureaucratic obstacles may prevent researchers and companies from submitting existing studies and conducting studies specifically for HMPC review. Overall, the significant reliance on external contributions highlights the necessity for the HMPC to proactively investigate new, relevant data and foster collaboration with interested parties to prevent overlooking important findings and reduce the risk of publishing incomplete or outdated monographs.

### Challenges of globalisation

To address the information gap on missing HMPs, explore further indications, and incorporate state of the art findings of more established phytopharmaceuticals into EMA monographs, EU member states need to provide more resources to conduct clinical trials on a broad spectrum of patients. Unfortunately, Europe falls short in conducting research on plant-based preparations in comparison to Asia and North America. As a result of increasing globalisation, almost 80% of all HMPs used in the European Union are produced outside of its borders [121]. An increasing number of studies supporting the marketing authorisation of phytopharmaceuticals are also being conducted in countries other than the EU [121], which proved to be an obstacle in this review. A total of 20 studies were excluded as they could not be retrieved in full text. In most cases, clinical trials were published in journals written in languages other than English and were often difficult to access. This circumstance occurred most frequently in Chinese trials (*n* = 9), followed by Russian (*n* = 4), Korean (*n* = 2) and Japanese (*n* = 1) studies. Additionally, there was no full-text version available for four clinical trials, despite being published in English.

International agreements were established to encourage the exchange of information, address common challenges, provide training in countries with less-developed regulatory systems, and advocate a global approach to HMP authorisation. To date, the EU has maintained agreements with Australia, Brazil, Canada, Israel, Japan, New Zealand, Switzerland, and the US [122]. The EMA also supports China and India in the implementation of good manufacturing practices and good clinical practices [123, 124]. However, it remains uncertain to what extent the aforementioned principles are implemented by the respective countries. The authorisation of HMPs based on long-standing use is particularly problematic given the unclear safety and efficacy, divergent regulatory requirements, and their use in special populations such as cancer patients. Simplified registration is intended to be used only where marketing authorisations cannot be obtained. Nevertheless, the results of this review show that 71.1% of all phytopharmaceuticals are licenced as THMPs due to a lack of clinical trials. This highlights a considerable need for research in this field so that THMPs can be approved as WEU-HMPs. Thus, a transition towards favouring the authorisation of well-established use HMPs based on evidence is justified.

In summary, member states of the European Union need to take action to promote research on HMPs and improve regulatory measures in the authorisation process. To ensure the safe and effective use of HMPs within the EU for a broad spectrum of patients, there is an urgent need for the following: 1) the completion of missing EMA monographs; 2) more clinical trials for all HMPs, especially those for which RCTs are not available; 3) periodic updates to incorporate the current state of knowledge; 4) the evaluation of phytopharmaceuticals without an EU standard; and 5) a shift towards pursuing marketing authorisations as well-established use HMPs.

### Limited statistical significance of HMPs in clinical trials

At present, a final evaluation on the efficacy of HMPs in cancer patients cannot be made due to the considerable proportion of non-significant endpoints and controversial data. Our comparative analysis did not provide sufficient evidence to draw any conclusion about the transferability of HMP indications or to make specific HMP recommendations for the treatment of adverse effects. These findings are consistent with the reasoning of the Oncology Guideline Program. It is noteworthy that even trials investigating at least one EMA-approved indication demonstrated inconclusive results. Examples include *cranberry* for urinary tract symptoms, *ginger* for nausea, and *lavender* for insomnia. Despite their inclusion in EMA monographs, these HMPs lack strong evidence of efficacy and were largely approved based on long-established traditional use. Similarly, studies on non-EMA indications showed non-significant results in 70% of cases, highlighting the limited evidence for HMPs.

### Limitations

This review is limited by the use of Medline and the Cochrane Library, which are databases that mainly cover HMPs of Western origin. To gain a deeper understanding of non-native plant-based preparations, follow-up projects should explore databases in other languages. 20 publications were either not retrievable or not available in full text. The majority of the articles were published in journals using languages other than English. Another potential obstacle may be the absence of a universal definition of patient-relevant outcomes (PROs), which can result in a heterogeneous selection of studies depending on the reviewer. This analysis defines the PRO as an improvement in treatment-related symptoms and an increase in QoL following the use of HMPs. Most of the scales used in this analysis interpret QoL as a comprehensive concept that includes physical, functional, emotional, and social dimensions. However, some studies only evaluated the physical component and were therefore unable to provide a holistic view of QoL [22, 25, 30, 33, 41, 66–68].

Our review is a comparative analysis, designed as an exploratory tool to provide an overview of the available evidence on the use of HMPs in managing treatment associated adverse effects in cancer patients. In this context, a p-value analysis was conducted to preliminarily review the efficacy of HMPs in comparison to placebo and other HMPs. While the p-value on its own cannot reflect the extent and clinical relevance of a phytopharmaceutical’s efficacy, it does provide the foundation for identifying studies with statistically significant results. Following consultation with all contributing authors, it was concluded that the generation of traditional forest plots was not feasible and reasonable due to two key factors: 1) the dataset is characterised by a substantial heterogeneity in terms of endpoints, study design and study population and 2) there is a lack of complete statistical data on the studies under review, particularly in regard to missing effect sizes. Instead, the p-value was investigated to provide an initial, structured assessment of the available evidence. This comparative analysis is not intended to serve as a substitute for a meta-analysis and does not allow for definitive conclusions to be drawn about the overall efficacy of HMPs in cancer patients. Rather, it was developed as a complementary approach to contextualise the current data and guide future research more effectively.

## Conclusion

For the first time, a common legal framework for the regulation of herbal medicinal products was established with the implementation of Directive 2001/83/EC and Directive 2004/24/EC of the European Parliament and Council. Harmonisation efforts have led to the development of EMA monographs for a growing number of HMPs that have successfully promoted cooperation between EU member states and standardised licensing processes, reflecting the European consensus. Scientific assessment and monitoring of pharmaceutical products are mainly regulated by the EMA, with most authorisation procedures conducted as traditional use registrations. Despite notable advancements, it remains uncertain whether the indications outlined in EMA monographs, primarily intended for personal use by non-cancer patients, can be readily applied to manage treatment-related adverse effects in cancer patients. On the one hand, there are considerable differences between the EMA monographs and the search results regarding the domains of application, both as a result of the research itself and the specifics of the EMA documents. On the other hand, there is a lack of sufficient data on the efficacy of HMPs for a wide range of indications. In particular, there is a shortage of high-quality RCTs investigating the evidence-based use of HMPs in patients with cancer. This literature review follows the approach of S3 guideline experts and concludes that, based on the currently available studies on phytopharmaceuticals, it is not possible to make any statement as to whether the use of HMPs to mitigate symptoms can be transferred from non-cancer patients to cancer patients. We confirmed the conclusion of the Oncology Guideline Program that there is insufficient evidence supporting the efficacy of most phytopharmaceuticals in addressing treatment-related adverse effects. Consequently, recommendations cannot be made for the majority of herbs as HMP studies in patients with cancer are lacking. To provide research-backed medical practices, build confidence in plant-based preparations, and maximise the full potential of phytopharmaceuticals, information gaps need to be addressed by standardising a systematic approach to the conduct of studies on HMPs. Initial efforts should be directed towards conducting RCTs on THMPs as the majority of phytopharmaceuticals were authorised via traditional use registration. The reliance on bibliographic data and full quality dossiers for the approval of THMPs - rather than clinical trials- has resulted in a shortage of satisfactory data demonstrating the efficacy of a substantial number of HMPs. Therefore, future clinical trials should reassess previous fields of application before exploring further HMP indications. Additionally, there is a need to update EMA documents at shorter intervals to incorporate the ever-growing knowledge on herbal medicine and facilitate scientific work in this complex field of research. A proactive revision strategy by the HMPC and the establishment of an international reporting network, are key elements to ensure the evidence-based and scientifically robust updating of EMA monographs.

## Supplementary Information

Supplementary Material 1: List of Included and Excluded Studies Sorted by HMP. Description of Data: overview of HMPs including availability of EMA monograph, indication according to EMA monograph and search strategy; list of inclusions and their study characteristics; list of exclusions and reasons for exclusion

Supplementary Material 2: List of Included Studies Sorted by EMA Indication Group and Non-EMA-Indication Group. Description of Data: Table 1 contains list of inclusions, their study characteristics and applicable EMA indication; Table 2 contains list of inclusions, their study characteristics and applicable non-EMA-indication.

Supplementary Material 3: Statistics. Description of Data: HMPs sorted by EMA monograph availability and compliance with inclusion criteria, data on PRISMA flow diagram, list of non-retrievable reports, distribution of reasons for exclusion, HMPs sorted by THMP and WEU-HMP, trials sorted by geographical distribution

Supplementary Material 4: Statistics. Description of Data: HMPs with respective endpoints, p-value of endpoints, analysis of individual significance and overall significance based on Excel formulas

## Data Availability

All data generated or analysed during this study are included in this published article and its supplementary information files.

## Abbreviations

EEA: European Economic Area
EFTA: European Free Trade Association
EMA: European Medicines Agency
EU: European Union
HMP: Herbal medicinal product
HMPC: Committee on Herbal Medicinal Products
MLWP: Monograph and List Working Party
PRO: Patient-relevant outcome
PRISMA: The Preferred Reporting Items for Systematic Reviews and Meta-Analyses
QoL: Quality of life
RCT: Randomised controlled trial
S3: Highest level of guideline production
THMP: Traditional use herbal medicinal product
WEU-HMP: Well-established use herbal medicinal products
