# Supplementary material for: Assessing indications for herbal medicinal products: a comparative analysis of EMA monographs and database records
Source: BMC Complement Med Ther. 2025 Apr 9;25:130. doi: 10.1186/s12906-025-04852-8 (PMC11980217; doi:10.1186/s12906-025-04852-8)
Supplement: Supplementary file 2 — Supplementary Material 2: List of Included Studies Sorted by EMA Indication Group and Non-EMA-Indication Group. Description of Data: Table 1 contains list of inclusions, their study characteristics and applicable EMA indication; Table 2 contains list of inclusions, their study characteristics and applicable non-EMA-indication. [file 12906_2025_4852_MOESM2_ESM.docx]

**Abbreviations**

BDI II: Beck Inventory Depression Scale II

BFI: Brief Fatigue Inventory

CFQ: Chalder Fatigue scale

CTCAE: Common Terminology Criteria for Adverse Events

DMA: dexamethasone + metoclopramide + aprepitant

DSSI: Dyspepsia Symptom Severity Index

EORTC: European Organisation for Research and Treatment of Cancer

EPIC: Expanded Prostate Index Composite

EQ-5D-5 L: EuroQol’s five dimension, 5-level scale

ESAS: Edmonton Symptom Assessment Scale

FACIT-F: Functional Assessment of Chronic Illness Therapy – Fatigue

FACT-G: Functional Assessment of Cancer Therapy-General

FLIE: Functional Living Index Emesis

HADS: Hospital Anxiety and Depression Scale

IC: interstitial cystitis symptom and problem scale

IPSS: International Prostate Symptom Score

MAT: MASCC Antiemesis Tool

MDASI: MD Anderson Symptom Inventory

MRS II: menopause rating scale II

PeNAT: Paediatric Nausea Assessment Tool

PG-SGA: Patient Generated Subjective Global Assessment

PSQI: Pittsburgh Sleep Quality Index

QoL: quality of life

RICAS: radiation induced cystitis assessment scale

RSCL: Rotterdam Symptom Checklist

RTOG: Radiation Therapy Oncology Group

VAS: visual analogue scale

Table 1. Characteristics of Selected Studies Included in Literature Review – EMA indication group

| HMP | first author and country | study design | study participants | experimental arms | primary endpoint | secondary endpoint | applicable EMA indication |
| --- | --- | --- | --- | --- | --- | --- | --- |
| black cohosh | Jacobson, USA, 2001 | RCT DB | 76 patients with breast cancer with completed primary therapy | - Intervention: Black Cohosh capsules (dose unknown) - Control: placebo | number and intensity of hot flashes using hot flash diary | LH and FSH levels | menopausal complaints such as hot flashes and profuse sweating in breast cancer patients |
| black cohosh | Pockaj, USA, 2006 | RCT DB | 132 patients with breast cancer, increased risk of breast cancer or refusal to take Estrogen | - Group 1: first Black cohosh capsules (2x20mg/day), then placebo - Group 2: first placebo, then Black Cohosh capsules (2x20mg/day) | hot flash score at beginning and end of the study | N/A | menopausal complaints such as hot flashes and profuse sweating in breast cancer patients |
| black cohosh | Pockaj, USA, 2004 | SAT | 21 patients with breast cancer, increased risk of breast cancer or refusal to take Estrogen | Black Cohosh capsules (Remifemin): 2x20mg/day | - average number of daily hot flashes per week - average hot flash score per week - proportion of patients with reduced hot flash scores | N/A | menopausal complaints such as hot flashes and profuse sweating in breast cancer patients |
| black cohosh | Rostock, Germany, 2011 | SAT | 50 patients with breast cancer receiving Tamoxifen | Black Cohosh capsules (Remifemin): 2x20mg/day | intensity of menopausal complaints using MRS II after 1, 3 and 6 months of therapy | - tumour history - co-medications - compliance - concomitant diseases - adverse effects - global assessment of effectiveness and tolerability | menopausal complaints such as hot flashes and profuse sweating in breast cancer patients |
| centella | Thanthong, Thailand, 2020 | RCT DB | 153 patients with breast cancer undergoing radiotherapy | - Group 1: no treatment - Group 2: moisturising cream - Group 3: Centella cream - Group 4: cucumber extract cream - Group 5: laurel clockvine cream | severity of radiation dermatitis using RTOG and EORTC scale | N/A | healing of minor wounds |

Table 1. Characteristics of Selected Studies Included in Literature Review – EMA indication group (continued)

| HMP | first author and country | study design | study participants | experimental arms | primary endpoint | secondary endpoint | applicable EMA indication |
| --- | --- | --- | --- | --- | --- | --- | --- |
| cranberry | Campbell, Canada, 2003 | RCT | 100 patients with prostate cancer undergoing radiotherapy | - Intervention: 354ml cranberry juice per day - Control: 354ml apple juice per day | maximum and maximum change in IPSS | use of additional symptomatic medication | mild recurrent lower urinary tract infections such as burning sensation during urination and/or frequent urination |
| cranberry | Cowan, UK, 2012 | RCT DB | 128 patients with bladder or cervix cancer undergoing radiotherapy | - Intervention: Cranberry juice (dose unknown) - Control: placebo juice (dose unknown) | development of urinary tract symptoms: increase in CTG grade or development of urinary tract infection | N/A | mild recurrent lower urinary tract infections such as burning sensation during urination and/or frequent urination |
| cranberry | Hamilton, New Zealand, 2015 | RCT DB | 40 patients with prostate cancer undergoing radiotherapy | - Intervention: Cranberry capsule (1x72mg/day) - Control: placebo capsule (1/day) | - frequency and severity of urinary tract symptoms using EPIC - degree of impairment due to urinary tract symptoms using EPIC | N/A | mild recurrent lower urinary tract infections such as burning sensation during urination and/or frequent urination |
| cranberry | Herst, New Zealand, 2020 | RCT DB | 101 patients with prostate cancer undergoing radiotherapy | - Intervention: Cranberry capsule (2x36mg/day) - Control: placebo capsule (2/day) | severity of acute cystitis due to radiotherapy using RTOG, IC and RICAS | N/A | mild recurrent lower urinary tract infections such as burning sensation during urination and/or frequent urination |
| ginger | Ansari, Iran, 2016 | RCT | 119 patients with breast cancer receiving doxorubicin | - Intervention: Ginger capsule (2x250mg/day) - Control: Placebo capsule with starch (2/day) | severity of nausea and vomiting using CTCAE | N/A | nausea and vomiting in motion sickness |
| ginger | Arslan, Turkey, 2015 | RCT | 60 patients with breast cancer receiving anthracycline | - Intervention: standard antiemetic drugs + 500mg Ginger powder dissolved in yoghurt - Control: standard antiemetic drugs + no treatment | - number of vomiting and retching episodes in diary - severity of nausea on a numerical 10-point scale | N/A | nausea and vomiting in motion sickness |
| ginger | Bossi, Italy, 2017 | RCT DB | 154 patients with solid cancer receiving cisplatin | - Intervention: Ginger capsule (4x40mg/day) - Control: placebo capsule (4/day) | incidence and severity of nausea using the VAS | - number of nausea episodes - FLIE scores - BFI scores | nausea and vomiting in motion sickness |

Table 1. Characteristics of Selected Studies Included in Literature Review – EMA indication group (continued)

| HMP | first author and country | study design | study participants | experimental arms | primary endpoint | secondary endpoint | applicable EMA indication |
| --- | --- | --- | --- | --- | --- | --- | --- |
| ginger | Crichton, Australia, 2024 | RCT DB | 103 patients with different types of solid cancer receiving chemotherapy | - Intervention: Ginger capsules (4x21mg/day) - Control: placebo capsules (4/day) | chemotherapy-induced nausea-related QoL | - vomiting related QoL - chemotherapy induced nausea and vomiting related QoL - health related QoL - anticipatory, acute, and delayed nausea and vomiting - fatigue - depression and anxiety - adverse effects - nutritional status | nausea and vomiting in motion sickness |
| ginger | Evans, USA, 2018 | RCT DB | 49 patients with different types of cancer receiving chemotherapy | - Intervention: Aromatherapy with ginger essential oil - Control 1: Water - Control 2: Johnson's Baby Shampoo | severity of nausea using PeNAT | N/A | nausea and vomiting in motion sickness |
| ginger | Khiewkhern, Thailand, 2013 | RCT SB | 66 patients with colorectal cancer receiving chemotherapy | - Intervention: massage with ginger and coconut oil - Control: standard supportive care | - leukocytes - neutrophils - lymphocytes - CD4 and CD8 cells - CD4/CD8 ratio | symptom severity scores for pain, fatigue, nausea, stress or anxiety and depression using a numerical 10-point rating scale | nausea and vomiting in motion sickness |
| ginger | Konmun, Thailand, 2017 | RCT DB | 81 patients with different types of solid cancers receiving chemotherapy | - Intervention: ginger capsules (2x10mg/day) - Control: placebo capsules (2/day) | complete response to ginger: no vomiting and no emergency treatment | - intensity of nausea and appetite using ESAS - QoL using FACT-G - adverse effects using CTCEA | nausea and vomiting in motion sickness |
| ginger | Li, China, 2018 | RCT DB | 146 patients with lung cancer receiving cisplatin | - Intervention: ginger capsules (2x250mg/day - Control: placebo capsules (2/day) | incidence and severity of acute and delayed nausea and vomiting using MAT | - QoL using FACT-G - adherence and adverse effects using self-reports | nausea and vomiting in motion sickness |
| ginger | Lua, Malaysia, 2015 | RCT SB | 60 patients with breast cancer receiving chemotherapy | - Intervention: aromatherapy with ginger oil - Control: placebo | - VAS nausea score - frequency of vomiting - health related QoL using EORTC QLQ-C30 | N/A | nausea and vomiting in motion sickness |

Table 1. Characteristics of Selected Studies Included in Literature Review – EMA indication group (continued)

| HMP | first author and country | study design | study participants | experimental arms | primary endpoint | secondary endpoint | applicable EMA indication |
| --- | --- | --- | --- | --- | --- | --- | --- |
| ginger | Manusirivithaya, Thailand, 2004 | RCT DB | 43 patients with breast cancer receiving cisplatin | - Intervention: ginger capsules (4x250mg/day) for 5 days - Control: placebo on day 1, metoclopramide on days 2 to 5 | - nausea using nausea score - adverse effects using general questioning | N/A | nausea and vomiting in motion sickness |
| ginger | Marx, Australia, 2020 | RCT DB | 33 patients with different types of cancer receiving chemotherapy | - Intervention: ginger capsules (4x15mg/day) - Control: placebo capsules | chemotherapy-induced nausea-related QoL using FLIE-5DR | - nutrition status using PG-SGA - anticipatory, acute, and delayed nausea and vomiting using MAT - fatigue using FACIT-F - depression and anxiety using HADS - global QoL using EQ-5D-5 L - health service use and costs - adverse events - adherence | nausea and vomiting in motion sickness |
| ginger | Panahi, Iran, 2012 | RCT OL | 78 patients with breast cancer receiving chemotherapy | - Intervention: 500mg ginger capsules + granisetron + dexamethasone - Control: granisetron + dexamethasone | prevalence, score and severity of nausea, vomiting, and retching using Rhodes Index | N/A | nausea and vomiting in motion sickness |
| ginger | Pillai, India, 2011 | RCT DB | 57 patients with sarcoma receiving cisplatin and doxorubicin | - Intervention: ginger capsules + ondansetron + dexamethasone - Control: placebo capsules + ondansetron + dexamethasone | incidence and severity of acute and delayed chemotherapy induced nausea and vomiting using ESAS und NCI | N/A | nausea and vomiting in motion sickness |
| ginger | Ryan, USA, 2012 | RCT DB | 576 patients with different types of cancer receiving chemotherapy | 1 ginger capsule contains 250mg ginger:   - Group 1: 3 placebo capsules twice per day - Group 2: 2 placebo capsules and 1 ginger capsule twice per day - Group 3: 1 placebo capsule and 2 ginger capsules twice per day - Group 4: 3 ginger capsules twice per day | dose and efficacy of ginger at reducing the severity of chemotherapy-induced nausea on Day 1 of chemotherapy | N/A | nausea and vomiting in motion sickness |

Table 1. Characteristics of Selected Studies Included in Literature Review – EMA indication group (continued)

| HMP | first author and country | study design | study participants | experimental arms | primary endpoint | secondary endpoint | applicable EMA indication |
| --- | --- | --- | --- | --- | --- | --- | --- |
| ginger | Sanaati, Iran, 2016 | RCT DB | 45 patients with breast cancer receiving chemotherapy | - Intervention 1: ginger capsules (2x500mg/day) + DMA - Intervention 2: chamomile capsules (2x500mg/day) + DMA - Control: DMA | - intensity of nausea - number of nausea episodes - number of vomiting episodes | N/A | nausea and vomiting in motion sickness |
| ginger | Santos, Spain, 2023 | RCT TB | 47 patients with cervical cancer receiving cisplatin | - Group 1: ginger capsules 250mg/day - Group 2: ginger capsules 500mg/day - Group 3: placebo capsules | nausea and vomiting using CTCAE | N/A | nausea and vomiting in motion sickness |
| ginger | Thamlikitkul, Thailand, 2017 | RCT DB | 34 patients with breast cancer receiving adriamycin and cyclophosphamide | Crossover study:   - Group 1: ginger capsules (2x500mg/day) in second cycle, placebo capsules in third cycle - Group 2: placebo capsules in second cycle, ginger capsules (2x500mg/day) in third cycle | reduction of nausea score using visual analogue scale | - vomiting incidence - rate of rescue medication use - incidence of chemotherapy dose reduction or delay - adverse events | nausea and vomiting in motion sickness |
| ginger | Williams, USA, 2022 | RCT SB | 147 patients with gastrointestinal, neuroendocrine, and skin cancer receiving chemotherapy | - Group 1: ginger essential oil inhalation - Group 2: German chamomile essential oil inhalation - Group 3: bergamot essential oil inhalation - Group 4: odourless control oil inhalation | appetite, anxiety, fatigue, nausea on 10-point Likert scale | acceptance and use of personal inhalation bottle | nausea and vomiting in motion sickness |
| ginger | Bhargava, Canada, 2020 | SAT | 15 patients with different types of cancer and anorexia-cachexia syndrome | ginger capsule (1x1650mg/day) | CRP, albumin, and ghrelin | - dyspepsia using DSSI - nausea using ESAS - weight loss, food intake, nutrition impact symptoms and functional capacity using PG-SGA | nausea and vomiting in motion sickness |
| ginger | de Queiroz, Brazil, 2024 | SAT | 35 patients with different types of cancer with no treatment options | ginger capsules (2x400mg/day) | QoL using EORTC QLQ-C30 | - anxiety and depression using HADS - fatigue using FACIT-F - adverse effects using CTCAE | nausea and vomiting in motion sickness |

Table 1. Characteristics of Selected Studies Included in Literature Review – EMA indication group (continued)

| HMP | first author and country | study design | study participants | experimental arms | primary endpoint | secondary endpoint | applicable EMA indication |
| --- | --- | --- | --- | --- | --- | --- | --- |
| guarana | da Costa Miranda, Brazil, 2009 | RCT DB | 36 patients with breast cancer undergoing radiotherapy | - Intervention: guarana (75mg/day) - Control: placebo | fatigue and depression using CFQ, BFI and BDI II | N/A | fatigue and sensation of weakness |
| guarana | de Oliveira Campos, Brazil, 2011 | RCT DB | 75 patients with breast cancer receiving chemotherapy | - Intervention: guarana (2x50mg/day) - Control: placebo | fatigue using FACIT-F global score | - FACT-ES global score - CFQ - BFI - PSQI - HADS | fatigue and sensation of weakness |
| guarana | del Giglio, Brazil, 2013 | RCT OL | 40 patients with different types of solid cancer receiving chemotherapy | - Intervention: guarana (2x37,5mg/day) - Control: placebo | fatigue using BFI | comparison of BFI scores to FACIT-F, CFQ, HADS and PSQI scores | fatigue and sensation of weakness |
| guarana | Palma, Brazil, 2016 | SAT | 18 patients with different types of cancer | guarana (2x50mg/day) | efficacy and safety of guarana i.e. increase in weight by at least 5% and increase in appetite by at least 3 points on the appetite scale | - FACT-G - MDASI | fatigue and sensation of weakness |
| linseed | Andersson, Sweden, 1995 | RCT SB | 20 patients with head and neck cancer undergoing radiotherapy | - Group 1: linseed extract in weeks 1 to 3, carboxymethyl cellulose preparation in weeks 5 to 7 - Group 2: carboxymethyl cellulose preparation in weeks 1 to 3, linseed extract in weeks 5 to 7 | mouth dryness | N/A | mild gastrointestinal discomfort |
| linseed | Lim, USA, 2021 | RCT | 20 patients with non-small cell lung cancer undergoing chemoradiotherapy | - Intervention: linseed (40g/day) - Control: no linseed | radiation pneumonitis using CTCAE | radiation esophagitis using CTCAE | mild gastrointestinal discomfort |

Table 1. Characteristics of Selected Studies Included in Literature Review – EMA indication group (continued)

| HMP | first author and country | study design | study participants | experimental arms | primary endpoint | secondary endpoint | applicable EMA indication |
| --- | --- | --- | --- | --- | --- | --- | --- |
| ginseng | Barton, USA, 2013 | RCT DB | 300 patients with different types of cancer | - Intervention: American ginseng capsules (2x2000mg/day) - Control: placebo capsules | fatigue using MFSI-SF | - profile of mood states (POMS) - BFI | symptoms of asthenia such as fatigue and weakness |
| ginseng | Barton, USA, 2010 | RCT DB | 175 patients with different types of cancer | - Intervention 1: American ginseng capsules (2x750mg/day) - Intervention 2: American ginseng capsules (2x1000mg/day) - Intervention 3: American ginseng capsules (2x2000mg/day) - Control: placebo capsules | fatigue using BFI | - Medical Outcome Scale Short Form 36 (SF36) - PSQI - Global Impression of Change - Linear Analogue Self-Assessment Scale - adverse effects using NRS and CTCAE | symptoms of asthenia such as fatigue and weakness |
| ginseng | Guglielmo, Italy, 2020 | RCT DB | 32 patients with head and neck cancer | - Intervention: American ginseng capsules (2x500mg/day) - Control: placebo capsules | fatigue using BFI | N/A | symptoms of asthenia such as fatigue and weakness |
| ginseng | Hamidian, Iran, 2023 | RCT DB | 40 patients with breast cancer receiving chemotherapy | - Intervention: ginseng capsules (1g/day) + chemotherapy - Control: placebo capsules + chemotherapy | health related QoL using FACT-B | N/A | symptoms of asthenia such as fatigue and weakness |
| ginseng | Jiang, China, 2017 | RCT | 60 patients with non-small lung cancer receiving chemotherapy | - Intervention: ginseng (300mg/day) + gemcitabine + cisplatin - Control: gemcitabine + cisplatin | - fatigue using Fatigue Symptom Inventory - anxiety using Self Rating Anxiety Scale - depression using Self Rating Depression Scale - Chinese Medicine Symptoms Score - Karnofsky Performance Status Scale - Functional Assessment of Cancer Therapy Lung - chemotherapy toxicity - biomarkers | N/A | symptoms of asthenia such as fatigue and weakness |

Table 1. Characteristics of Selected Studies Included in Literature Review – EMA indication group (continued)

| HMP | first author and country | study design | study participants | experimental arms | primary endpoint | secondary endpoint | applicable EMA indication |
| --- | --- | --- | --- | --- | --- | --- | --- |
| ginseng | Kim, South Korea, 2017 | RCT DB | 30 patients with epithelial ovarian cancer receiving chemotherapy | - Interventions: red ginseng capsules (3x 2 500mg capsules/day) - Control: placebo capsules | - toxicity using CTCAE and blood analysis - health related QoL using EORTC QLQ-C30, BFI, HADS and MOS-SS - survival after adjuvant chemotherapy | N/A | symptoms of asthenia such as fatigue and weakness |
| ginseng | Kim, South Korea, 2020 | RCT DB | 409 patients with colorectal cancer receiving mFOLFOX-6 | - Intervention: ginseng capsules (2x 2 500mg capsules/day) - Control: placebo capsules | fatigue using BFI | - fatigue related QoL using FACIT-F - stress using Stress Index - adverse effects using CTCAE | symptoms of asthenia such as fatigue and weakness |
| ginseng | Yennurajalingam, USA, 2017 | RCT DB | 112 patients with different types of cancer receiving chemotherapy | - Intervention: ginseng capsules (2x400mg/day) - Control: placebo capsules | fatigue using FACIT-F | - fatigue using ESAS - anxiety and depression using HADS - patient reported treatment benefit using (GSE) | symptoms of asthenia such as fatigue and weakness |
| ginseng | Yennurajalingam, USA, 2015 | SAT | 24 patients with different types of cancer receiving chemotherapy | ginseng capsules (2x400mg/day) | - fatigue using FACIT-F - symptoms of cancer using ESAS - anxiety and depression using HADS - patient reported treatment benefit using GSE - adverse effects using CTCAE | N/A | symptoms of asthenia such as fatigue and weakness |

Table 1. Characteristics of Selected Studies Included in Literature Review – EMA indication group (continued)

| HMP | first author and country | study design | study participants | experimental arms | primary endpoint | secondary endpoint | applicable EMA indication |
| --- | --- | --- | --- | --- | --- | --- | --- |
| green tea | Liu, China, 2021 | RCT OL | 77 patients with gastric cancer undergoing subtotal distal gastrectomy | - Intervention: 500ml green tea on first postoperative day, 1000ml green tea on consecutive days - Control: 500ml water on first postoperative day, 1000ml water on consecutive days | recovery of gastrointestinal function measured by first flatus, time to first bowel motion and time to tolerate solid food | - incidence of postoperative complications - nausea, vomiting, diarrhoea, and bloating - postoperative hospital stay - postoperative pain using VAS - postoperative fatigue using Fatigue Score Model | symptoms of asthenia such as fatigue and weakness |
| green tea | Choan, Canada, 2005 | SAT | 19 patients with hormone refractory prostate cancer | green tea capsules (2x250mg/day) | prostate cancer progression: 1) relative PSA increase of more than 25% or 2) evidence of radiologic progression | toxicity using self-reporting form and visits by attending physicians: among others is fatigue | symptoms of asthenia such as fatigue and weakness |
| green tea | Jatoi, USA, 2003 | SAT | 42 patients with asymptomatic androgen independent metastatic prostate cancer | green tea capsules (6x1mg/day) | - tumour response defined as decline of PSA level of at least 50% - toxicity using CTCAE: such as fatigue, nausea, insomnia | N/A | symptoms of asthenia such as fatigue and weakness |
| green tea | Pisters, USA, 2001 | SAT | 49 patients with different types of solid cancer | green tea capsules: 110mg, 200mg or 270mg   - cohorts 1 to 7: 1x/day - cohorts 8 to 10: 3x/day | - maximum tolerated dose - toxicity using NCI: among them is fatigue - pharmacology | N/A | symptoms of asthenia such as fatigue and weakness |
| lavender flower/ lavender oil | Beyliklioğlu, Turkey, 2019 | RCT | 80 patients with breast cancer undergoing surgery | - Intervention: lavender oil inhalation on surgery day - Control: no inhalation | anxiety using STAI | N/A | mild symptoms of mental stress and exhaustion and to aid sleep |
| lavender flower/ lavender oil | Khamis, Egypt, 2023 | RCT DB | 100 patients with different types of cancer receiving palliative care | - Group 1: standard hospital treatment - Group 2: hand massage with carrier oil (almond oil) - Group 3: hand massage with lavender oil - Group 4: hand massage with lavender oil + inhalation of lavender oil | QoL using RSCL among them sleep quality | N/A | mild symptoms of mental stress and exhaustion and to aid sleep |

Table 1. Characteristics of Selected Studies Included in Literature Review – EMA indication group (continued)

| HMP | first author and country | study design | study participants | experimental arms | primary endpoint | secondary endpoint | applicable EMA indication |
| --- | --- | --- | --- | --- | --- | --- | --- |
| lavender flower/ lavender oil | Ozkaraman, Turkey, 2018 | RCT DB | 70 patients with different types of cancer receiving chemotherapy | - Group 1: lavender oil inhalation - Group 2: tea tree oil inhalation - Group 3: no treatment | - STAI - PSQI | N/A | mild symptoms of mental stress and exhaustion and to aid sleep |
| lavender flower/ lavender oil | Şahin, Turkey, 2023 | RCT blinded outcome evaluation | 45 patients with different types of cancer | - Group 1: foot soak - Group 2: lavender oil inhalation - Group 3: foot soak and lavender oil inhalation | severity of insomnia using insomnia severity index (ISI) and MD Anderson Symptom Inventory (MDASI) | N/A | mild symptoms of mental stress and exhaustion and to aid sleep |
| lavender flower/ lavender oil | Shammas, USA, 2021 | RCT SB | 49 patients with breast cancer undergoing surgery | - Intervention: lavender oil - Control: placebo (coconut oil) | - perioperative stress, anxiety and depression using HADS - pain using VAS - sleep using Richards Campbell Sleep Questionnaire | N/A | mild symptoms of mental stress and exhaustion and to aid sleep |
| lavender flower/ lavender oil | Soden, UK, 2004 | RCT DB | 42 patients with different types of cancer receiving palliative care | - Group 1: massage with lavender oil and carrier oil - Group 2: massage with carrier oil - Group 3: no massage | pain intensity using VAS | - sleep quality using Verran and Snyder Halpern sleep scale - anxiety and depression using HADS - QoL using RSCL | mild symptoms of mental stress and exhaustion and to aid sleep |
| lavender flower/ lavender oil | Kohara, Japan, 2004 | SAT | 20 patients with different types of cancer | aromatherapy with lavender oil, foot soak and reflexology treatment with lavender rich jojoba oil | fatigue using cancer fatigue scale (CFS) | N/A | mild symptoms of mental stress and exhaustion and to aid sleep |
| roman chamomile flower | Wilkinson, UK, 1999 | RCT | 87 patients with different types of cancer receiving palliative care | - Intervention: massage with roman chamomile essential oil and carrier oil - Control: massage with carrier oil | - QoL using RSCL among them abdominal pain, obstipation, and diarrhoea - anxiety using STAI - semi structured questionnaire | N/A | mild, spasmodic gastrointestinal complaints such as bloating and flatulence |

Table 2. Characteristics of Selected Studies Included in Literature Review – non-EMA-indication group

| HMP | first author, country, year | study design | study participants | experimental arms | primary endpoint | secondary endpoint | applicable non-EMA indication |
| --- | --- | --- | --- | --- | --- | --- | --- |
| aloe vera | Alkhouli, Syria, 2021 | RCT DB | 26 patients with acute lymphoblastic leukaemia receiving chemotherapy | - Intervention: aloe vera - Control: sodium bicarbonate 5% | chemotherapy induced oral mucositis using WHO grading scale | N/A | oral mucositis |
| aloe vera | Heggie, Australia, 2022 | RCT DB | 208 patients with breast cancer undergoing radiotherapy | - Intervention: aloe vera gel 3 times/day - Control: aqueous cream 3 times/day | acute skin toxicity using Form B | impact of breast size, smoking habits, and number of postoperative drains on severity of skin damage | radiation dermatitis |
| aloe vera | Hoopfer, Canada, 2015 | RCT DB | 237 patients with breast cancer undergoing radiotherapy | - Intervention: aloe vera cream - Control 1: placebo cream - Control 2: dry powder skin care | acute skin toxicity using Catterall scale (CSSP) | N/A | radiation dermatitis |
| aloe vera | Lissoni, Italy, 1998 | RCT | 50 patients with different types of cancer | - Intervention: melatonin + aloe vera - Control: melatonin | tumour progression using WHO criteria and 1 year survival rate | N/A | cancer progression |
| aloe vera | Lissoni, Italy, 2009 | RCT | 240 patients with different types of cancer receiving chemotherapy | - Intervention: chemotherapy + aloe vera - Control: chemotherapy | tumour progression using WHO criteria | N/A | cancer progression |
| aloe vera | Momm, Germany, 2005 | RCT | 120 patients with head and neck cancer undergoing radiotherapy | - Group 1: aloe vera gel - Group 2: carmellose spray - Group 3: rape oil - Group 4: mucin spray | radiation induced mouth dryness using total score of the questionnaire | score of single items on the questionnaire and two additional questions | mouth dryness |
| aloe vera | Sahebjamee, Iran, 2015 | RCT TB | 26 patients with head and neck cancer undergoing radiotherapy | - Intervention: aloe vera mouthwash - Control: benzydamine mouthwash | radiation induced oral mucositis using WHO grading system | N/A | oral mucositis |
| aloe vera | Sahebnasagh, Iran, 2020 | RCT DB | 42 patients with pelvic floor cancer undergoing radiotherapy | - Intervention: aloe vera 3% topical ointment - Control: placebo topical ointment | radiation induced proctitis using RTOG and clinical examination | - QoL - psychosocial status using HADS - CRP | proctitis |
| aloe vera | Sahebnasagh, Iran, 2022 | RCT DB | 20 patients with colorectal cancer undergoing radiotherapy | - Intervention: aloe vera 3% topical ointment - Control: placebo topical ointment | radiation induced proctitis using RTOG and clinical examination | - QoL - psychosocial status using HADS - CRP | proctitis |

Table 2. Characteristics of Selected Studies Included in Literature Review – non-EMA-indication group (continued)

| HMP | first author, country, year | study design | study participants | experimental arms | primary endpoint | secondary endpoint | applicable non-EMA indication |
| --- | --- | --- | --- | --- | --- | --- | --- |
| aloe vera | Su, USA, 2004 | RCT DB | 58 patients with head and neck cancer undergoing radiotherapy | - Intervention: aloe vera solution containing 94,5% aloe vera, 5% pear juice, 0,4% lemon lime flavour and 0,1% citric acid - Control: placebo solution containing 94,5% water, 5% pear juice, 0,4% lemon lime flavour and 0,1% citric acid | incidence and duration of mucositis | - QoL - weight loss - use of pain medications - need for intravenous hydration - occurrence of oral infections - duration of treatment interruptions | oral mucositis |
| aloe vera | Tungkasamit, Thailand, 2022 | RCT DB | 120 patients with head and neck cancer undergoing chemoradiotherapy | - Intervention: aloe vera gel - Control: placebo gel | severity of radiation induced dermatitis using radiation-induced skin reaction assessment scale (RISRAS) | N/A | radiation dermatitis |
| aloe vera | Williams, USA, 1996 | RCT DB | patients with breast cancer undergoing radiotherapy: 194 patients in first, 108 patients in second trial | first trial:   - Intervention: aloe vera gel - Control: placebo gel   second trial:   - Intervention: aloe vera gel - Control: no treatment | radiation induced dermatitis using maximum severity score | N/A | radiation dermatitis |
| aloe vera | Nyström, Sweden, 2007 | SAT | 50 patients with breast cancer undergoing radiotherapy | aloe vera gel, Essex lotion and no lotion on different radiation sites | radiation dermatitis using Near Infrared Spectroscopy, Digital Colour Photography und Laser Doppler | N/A | radiation dermatitis |
| evening primrose oil | van der Merwe, South Africa, 1990 | RCT DB | 62 patients with primary liver cancer | - Intervention: evening primrose oil capsules (36x500mg/day) - Control: placebo capsules | - liver size - GGT levels - mean survival time | - complete blood count - haemoglobin - urea - electrolytes - albumin - AP - AFP | liver function |
| garlic | Gatt, Israel, 2015 | RCT DB | 95 patients with malignant haematological disease receiving chemotherapy | - Intervention: garlic extract tablet (2x450mg/day) - Control: placebo tablet | - onset of fever > 38°C - time to first fever during study time period | - length of each febrile episode - number of recurrent febrile episodes | fever |

Table 2. Characteristics of Selected Studies Included in Literature Review – non-EMA-indication group (continued)

| HMP | first author, country, year | study design | study participants | experimental arms | primary endpoint | secondary endpoint | applicable non-EMA indication |
| --- | --- | --- | --- | --- | --- | --- | --- |
| peppermint oil | Efe Ertürk, Turkey, 2021 | Quasi RCT OL | 80 patients with different types of cancer receiving chemotherapy | - Intervention: peppermint oil (3x1 drop/day) + standard antiemetic drugs - Control: standard antiemetic drugs   standard antiemetic drugs:   - before chemotherapy: Granisetron + dexamethasone + metoclopramide - after chemotherapy: ondansetron + metoclopramide | - severity of nausea using VAS - Index of Nausea, Vomiting, and Retching (INVR) | patient opinions forms on aromatherapy practice | nausea, vomiting and retching |
| rhubarb | Yu, China, 2008 | RCT DB | 74 patients with lung cancer undergoing radiotherapy | - Intervention: rhubarb extract (20mg/kg/day) + 3D conformal radiation therapy (3D-CRT) - Control: placebo + 3D conformal radiation therapy (3D-CRT) | severity of radiation induced lung toxicity using Lent Soma scale by RTOG and EORTC | - pulmonary function - TGF-$\beta$1 - IL-6 | radiation induced lung toxicity |
| tea tree oil | Ozkaraman, Turkey, 2018 | RCT DB | 70 patients with different types of cancer receiving chemotherapy | - Group 1: lavender oil inhalation - Group 2: tea tree oil inhalation - Group 3: no treatment | - STAI - PSQ | N/A | anxiety and sleep quality |
| turmeric | Howells, UK, 2019 | RCT OL | 24 patients with metastatic colorectal cancer receiving chemotherapy | - Group 1: FOLFOX - Group 2: FOLFOX + curcumin (2g/day) | - safety using CTCAE - efficacy using progression free survival and overall survival | - QoL using EORTC QLQ-C30 - neurotoxicity using FACT-GOG-NTX - CXCL1 using ELISA | QoL |
| turmeric | Kia, Iran, 2021 | RCT DB | 50 patients with different types of cancer receiving chemotherapy | - Intervention: curcumin nanomicelle capsules (2x80mg/day) - Control: placebo capsules (2/day) | - severity of oral mucositis using WHO mucositis scale - pain using NRS | N/A | oral mucositis |
| turmeric | Palatty, India, 2014 | RCT SB | 46 patients with head and neck cancer undergoing radiotherapy | - Group 1: Johnson’s Baby Oil - Group 2: Vicco turmeric cream | severity of radiation dermatitis using RTOG | N/A | radiation dermatitis |
| turmeric | Rao, India, 2014 | RCT SB | 79 patients with head and neck cancer undergoing radiotherapy or receiving chemoradiotherapy | - Intervention: turmeric gargle (6/day) - Control: povidone iodine gargle (6/day) | incidence of mucositis every week using RTOG | - incidence of treatment breaks - loss of scheduled treatment days - decrease in body weight | oral mucositis |

Table 2. Characteristics of Selected Studies Included in Literature Review – non-EMA-indication group (continued)

| HMP | first author, country, year | study design | study participants | experimental arms | primary endpoint | secondary endpoint | applicable non-EMA indication |
| --- | --- | --- | --- | --- | --- | --- | --- |
| turmeric | Ryan Wolf, USA, 2018 | RCT DB | 686 patients with breast cancer undergoing radiotherapy | - Intervention: curcumin capsules (3x four 500mg capsules/day) - Control: placebo capsules | severity of radiation dermatitis using RDS scale | - presence of moist desquamation - pain at radiotherapy site using SF-MPQ - skin related QoL using Skindex-29 - adverse effects using Symptom Inventory (SI) | radiation dermatitis |
| turmeric | Shah, India, 2020 | RCT TB | 74 patients with head and neck cancer undergoing radiotherapy | - Intervention: 0,1% curcumin mouthwash - Control: 0,15% benzydamine mouthwash | risk of onset, effect on progression and severity of radiation induced oral mucositis using WHO criteria | safety of mouthwashes | oral mucositis |
| turmeric | Soni, India, 2022 | RCT DB | 60 patients with different types of oral cavity cancer receiving chemoradiotherapy | - Group 1: turmeric capsules (2x500mg/day) - Group 2: turmeric capsules (3x500mg/day) - Group 3: placebo capsules (3/day) | incidence and severity of chemoradiotherapy induced oral mucositis using CTCAE | - incidence and severity of dysphagia - oral pain - dermatitis - significant weight loss - compliance | oral mucositis |
| turmeric | Thomas, India, 2023 | RCT | 92 patients with head and neck cancer receiving chemoradiotherapy | - Intervention: turmeric mouthwash - Control: benzydamine mouthwash | - oral health status using Oral Health Assessment Tool - severity of oral mucositis using WHO toxicity criteria - oral dysfunction using oral mucositis symptom scale and xerostomia short-form inventory | N/A | oral mucositis |
| yarrow | Foucré, Germany, 2022 | RCT OL | 20 patients with different types of cancer undergoing radiotherapy for bone or brain metastasis | - Intervention: yarrow liver compress - Control: no treatment | fatigue using heart rate variability | N/A | fatigue |
| yarrow | Ghadjar, Germany, 2021 | RCT OL | 39 patients with different types of cancer undergoing radiotherapy for bone or brain metastasis | - Intervention: yarrow liver compress - Control: no treatment | fatigue using MFI-20 | - psychological distress using NCCN - QoL using EORTC QLQ-C30 - qualitative analysis using VAS | fatigue |

Table 2. Characteristics of Selected Studies Included in Literature Review – non-EMA-indication group (continued)

| HMP | first author, country, year | study design | study participants | experimental arms | primary endpoint | secondary endpoint | applicable non-EMA indication |
| --- | --- | --- | --- | --- | --- | --- | --- |
| ginseng | Chung, South Korea, 2021 | RCT DB | 55 patients with different types of gynaecological cancer | - Intervention: Korean ginseng capsules (3g/day) - Control: placebo capsules | menopausal symptoms using menopause rating scale (MRS) | N/A | menopausal symptoms |
| ginseng | Kim, South Korea, 2006 | RCT DB | 53 patients with different types of cancer | - Interventions: ginseng capsules (3x1000mg/day) - Control: placebo capsules | QoL using WHO Quality of Life Assessment (WHO QOL BREF) and General Health Questionnaire 12 (GHQ-12) | N/A | QoL |
| ginseng | Xie, China, 2001 | RCT | 131 patients with nasopharyngeal cancer undergoing radiotherapy | - Intervention: ginseng polysaccharide + radiotherapy - Control: radiotherapy | - local cancer remission - 1-year overall survival rate - survival rate without cancer - survival rate without distant metastases | - changes in T lymphocyte subgroups - activity of natural killer cells - activity of lymphokine activated killer cells | cancer progression/ survival |
| green tea | Liao, Taiwan, 2021 | RCT SB | 61 patients with oral cancer undergoing cancer treatment | - Intervention: green tea mouthwash (2x/day) - Control: water mouthwash (2x/day) | oral health status using Oral Assessment guide | N/A | oral mucositis |
| green tea | Trudel, Canada, 2013 | SAT | 16 patients with serous or endometrioid ovarian cancer FIGO stage 3 and 4 | green tea 500ml/day | absence of recurrence during follow-up of 18 months | adverse effects using CTCAE | cancer recurrence |
| lavender flower/ lavender oil | Duluklu, Turkey, 2019 | RCT SB | 30 patients with colorectal cancer undergoing colostomy surgery | - Intervention: lavender oil in colostomy bag - Control: regular colostomy bag care | elimination of odour related to ostomy bag | - QoL using stoma quality of life scale (SQOLS) - ostomy adjustments using ostomy adjustment inventory-23 (OAI-23) | odour management of colostomy bag |
| roman chamomile flower | Dos Reis, Brazil, 2016 | RCT SB | 38 patients with gastric or colorectal cancer receiving 5-FU and leucovorin | - Intervention: cryotherapy made of chamomile infusion - Control: cryotherapy made only with water | occurrence of oral mucositis using WHO scale: evaluation by dentist on days 8, 15, and 22 after first day of chemotherapy | - intensity of oral mucositis using WHO scale - mouth pain using numerical 10-point rating scale - presence of erythema, ulceration, mouth dryness, bleeding, and dietary category | oral mucositis |

Table 2. Characteristics of Selected Studies Included in Literature Review – non-EMA-indication group (continued)

| HMP | first author, country, year | study design | study participants | experimental arms | primary endpoint | secondary endpoint | applicable non-EMA indication |
| --- | --- | --- | --- | --- | --- | --- | --- |
| roman chamomile flower | Ferreira, Brazil, 2020 | RCT DB | 48 patients with head and neck cancer undergoing radiotherapy | - Intervention: chamomile gel 8,35% (3x/day)   Control: urea cream (3x/day) | - presence of skin toxicity - severity of radiation dermatitis using RTOG | erythema onset time | radiation dermatitis |
| roman chamomile flower | Garbuio, Brazil, 2022 | RCT DB | 54 patients with breast cancer receiving chemoradiotherapy | - Intervention: chamomile microparticles (1x/day) - Control: placebo (1x/day) | development of radiation dermatitis grade using incidence and time of onset using RTOG | - incidence and time for development of radiation dermatitis grad 2 or higher using RTOG - skin related QoL using Skindex 16 - pain, itching and burning using VAS | radiation dermatitis |
| roman chamomile flower | Maiche, Finland, 1991 | RCT DB | 48 patients with breast cancer undergoing radiotherapy | - Intervention: chamomile cream (2x/day) - Control: almond ointment (2x/day) | severity of radiation dermatitis using own scale | N/A | radiation dermatitis |
| roman chamomile flower | Sanaati, Iran, 2016 | RCT DB | 45 patients with breast cancer receiving chemotherapy | - Intervention 1: ginger capsules (2x500mg/day) + DMA - Intervention 2: chamomile capsules (2x500mg/day) + DMA - Control: DMA = dexamethasone + metoclopramide + aprepitant | - intensity of nausea - number of nausea episodes - number of vomiting episodes | N/A | nausea and vomiting |
| roman chamomile flower | Williams, USA, 2022 | RCT SB | 147 patients with gastrointestinal, neuroendocrine, and skin cancer receiving chemotherapy | - Group 1: ginger essential oil inhalation - Group 2: German chamomile essential oil inhalation - Group 3: bergamot essential oil inhalation   Group 4: odourless control oil inhalation | appetite, anxiety, fatigue, nausea on 10-point Likert scale | acceptance and use of personal inhalation bottle | nausea and vomiting |
